# Quantitative detection and genetic characterization of thermotolerant *Campylobacter* spp. in fresh chicken meats at retail in Japan

**DOI:** 10.3389/fmicb.2022.1014212

**Published:** 2022-10-10

**Authors:** Hiroshi Asakura, Shiori Yamamoto, Kazuhiro Yamada, Jun Kawase, Hiromi Nakamura, Kou-ichiro Abe, Yoshimasa Sasaki, Tetsuya Ikeda, Ryohei Nomoto

**Affiliations:** ^1^Division of Biomedical Food Research, National Institute of Health Sciences, Kawasaki, Kanagawa, Japan; ^2^Department of Microbiology and Medical Zoology, Aichi Prefectural Institute of Public Health, Nagoya, Aichi, Japan; ^3^Department of Bacteriology, Shimane Prefectural Institute of Public Health and Environmental Science, Matsue, Shimane, Japan; ^4^Department of Microbiology, Osaka Institute of Public Health, Osaka, Japan; ^5^Kawasaki City Institute of Public Health, Kawasaki, Kanagawa, Japan; ^6^Department of Infectious Diseases, Hokkaido Institute of Public Health, Sapporo, Hokkaido, Japan; ^7^Department of Infectious Diseases, Kobe Institute of Health, Kobe, Hyogo, Japan

**Keywords:** retailed poultry meat, contamination level, antimicrobial resistance, whole-genome sequencing, *Campylobacter jejuni*/*coli*

## Abstract

*Campylobacter jejuni* and *C. coli* are one of the leading causes of gastrointestinal illnesses, and which are considered to be transmitted to humans mainly from chicken meats. Considering the less availability of quantitative contamination data in the retail chicken meats in Japan, 510 fresh chicken meats retailed at five distinct regions in Japan between June 2019 and March 2021 were examined. The quantitative testing resulted that 45.7% of the samples (254/510) were positive at mean ± standard deviation of 1.15 ± 1.03 logCFU/g, whereas 43 samples (8.4%) exceeded 3.0 logCFU/g. Seasonal comparison revealed increased bacterial counts in fall compared with spring and summer. As for the chicken slaughter age, those slaughtered at >75 days old were less contaminated than those at <75 days old. Genome sequencing analyses of 111 representative *C. jejuni* isolates resulted in the detection of three antimicrobial resistance genes (*gyrA* substitution T86I, *tetO* and *blaOXA-61*) at 25.2, 27.9 and 42.3%, respectively. In silico MLST analysis revealed the predominance of sequence types (ST)-21 clonal complex (CC), followed by ST-45CC and ST-464CC. The single nucleotide polymorphism (SNP)-based phylogenetic tree largely classified the sequenced *C. jejuni* isolates into two clusters (I and II), where all *C. jejuni* from highly contaminated samples (STs-21CC, -22CC and -45CC) belonged to cluster I, independent of both season and slaughter age. To our knowledge, this is the first example to study the current status of *Campylobacter* contamination levels in fresh chicken meats retailed in Japan. Our data would be contributable to future quantitative microbial risk assessment, to establish effective control measures for campylobacteriosis.

## Introduction

*Campylobacter jejuni* and *C. coli* are among the most reported foodborne pathogens to cause human gastrointestinal disease globally [Geissler et al., 2017; European Food Safety Authority (EFSA) and European Centre for Disease Prevention and Control (ECDC), 2021; Sher et al., 2021]. Human campylobacteriosis is usually linked to the consumption of poultry meat, particularly in fresh portioned and whole chicken meat products (Kaakoush et al., 2015; Batz et al., 2021), highlighting the necessity to control this pathogen along the poultry production chain. The focus on the risk of *Campylobacter* contamination from poultry meats is due to their high international consumption, estimated at 13.9 kg *per capita* in 2015–2017 and expected to increase up to 14.6 kg in 2027 [Food and Agriculture Organization (FAO) and Organization for Economic Co-operation and Development (OECD), 2018]. Similarly, the slaughtered weight ranges of poultry meats in Japan have been increasing, as previously reported, from 2,171,905 tonnes in 2016 to 2,331,650 tonnes in 2020 [Ministry of Agriculture, Forestry and Fisheries (MAFF), 2021]. To evaluate the risk associated with poultry consumption and its reduction measures, quantitative microbial risk assessment (QMRA) is currently employed as a structured approach that enables the estimation of the illness probability in a population; QMRA consists of the detection of the contamination levels of human/animal-related pathogens at any process, which may jeopardize human health (Chapman et al., 2016; Mikkelä et al., 2016).

In 2020, 20.5% (182/887) of the total cases of foodborne gastrointestinal illness in Japan were caused by campylobacteriosis [Ministry of Health, Labour and Welfare (MHLW), 2021]. Moreover, our previous study estimated that the source of nearly 80.3% of the detected campylobacteriosis was chicken meats (Kumagai et al., 2020). In June 2021, the MHLW in Japan adopted the national Food Hygiene Act [Ministry of Health, Labour and Welfare of Japan (MHLW), 2020], which enables all poultry slaughterhouses to apply HACCP principles, including microbiological monitoring system with validation and verification programmes [Ministry of Health, Labour and Welfare of Japan (MHLW), 2020]. However, there is a lack of a nationwide monitoring system for poultry meat products at the retail level in Japan.

European agency has suggested QMRA as a tool to reduce the impact of human campylobacteriosis emerging from broiler meat [EFSA Panel on Biological Hazards (BIOHAZ), 2010], in which the use of the QMRA model was suggested to estimate the impact of the presence of *Campylobacter* spp. in the broiler meat chain on human campylobacteriosis. Furthermore, the model used available quantitative data to rank/categorize the designated intervention approaches along the farm-to-fork continuum. In this context, some countries have already initiated the acquisition of a quantitative baseline data regarding *Campylobacter* contamination mainly at the poultry slaughter level (Bai et al., 2014; Lynch et al., 2022), while these data at the retail level were limited [Public Health England (PHE), 2019]. The Food Safety Commission of Japan (FSCJ), a national organization for risk assessment, recently revised the risk profiles of *C. jejuni* and *C. coli* in chicken meats and advised for the acquisition of such quantitative data through standardized microbiological methods [Food Safety Commission of Japan (FSCJ), 2021].

Given the background, this study aimed to quantitatively detect thermotolerant *Campylobacter* spp. from a total of 510 chicken thigh meat portions retailed in five distinct regions across Japan. Combined with their epidemiological history, possible factors influencing the levels of *Campylobacter* contamination were analyzed and discussed. Moreover, we used whole genome sequencing on the *C. jejuni* isolates.

## Materials and methods

### Food samples

Fresh-chilled chicken thigh meat products (total *n* = 510) were purchased from retail markets in five different regions (A–E; Supplementary Figure 1) across Japan between June 2019 and May 2021. The samples were transported at <10°C and subjected to microbiological examination within 3 h.

### Enumeration of thermotolerant *Campylobacter* spp.

Considering the fact that greater dissemination of the thermophilic *Campylobacter* spp. relies on the skin rather than on the underlying muscle parts in retail chicken meat portions (Hansson et al., 2015), we selected skin parts as target for the bacterial testing. We quantitatively detected thermotolerant *Campylobacter* spp. from chicken meats essentially based on ISO 10272-2:2017 [International Organization for Standardization (ISO), 2017]. In brief, from each chicken meat product, a sample of 25 g of skin was cut using sterile scissors and tweezers, followed by morcellation and homogenisation for 1 min in 100 ml of buffered peptone water (BPW; Oxoid, Hampshire, United Kingdom). We spread aliquots of 1-mL BPW suspension with its serial dilutions on modified charcoal cefoperazone deoxycholate agar (mCCDA) plates (Oxoid), which were incubated at 42°C for 48 h under microaerophilic condition using the AnaeroPack-MicroAero system (Mitsubishi Gas Chemicals, Tokyo, Japan). We counted the number of colonies, where at least five typical or suspected colonies per plate were subjected to real-time PCR to confirm the presence of *C. jejuni* or *C. coli*, as previously described (Kawase et al., 2016). Finally, we used this number to calculate the total bacterial count in each sample. Because the theoretical limit of detection (LOD) of the aforementioned assay was estimated at 0.70 logCFU/g, we adopted 1/2 LOD (=0.35 logCFU/g) for *Campylobacter*-negative samples. After the test confirmation, the obtained *C. jejuni* isolates from the samples in regions B, C and E were microaerobically grown on Mueller–Hinton agar plates (Becton Dickinson, Franklin Lakes, NJ, United States) at 37°C for 20 h and stored in 15% glycerol in Tryptic Soy Broth (Becton Dickinson) at −80°C until further use. In this study, we did not use qualitative method for the detection and isolation of thermotolerant *Campylobacter* spp.

### DNA extraction

A total of 111 representatives of *C. jejuni* isolates from chicken meats at regions B, C and E were arbitrarily selected to include highly contaminated sample origin (>3.0 logCFU/g). The bacterial isolates were grown on Mueller–Hinton agar (Becton Dickinson) at 37°C for 20 h under microaerophilic conditions using the AnaeroPack-MicroAero system, followed by centrifugation at 4,000× *g* for 5 min. Accordingly, we extracted DNA from the bacterial pellets using the Maxwell Blood DNA Kit (Promega, Madison, WI, USA). We measured the concentration and quality of the extracted DNA on a TapeStation 4,150 system (Agilent Technologies, Santa Clara, CA, USA). The samples were stored at −80°C until further use.

### Whole-genome sequencing analysis

To construct libraries of DNA, we used 1 μg of each of the bacterial extracts with the Ion Xpress Plus Fragment Library Kit (Thermo Fisher Scientific, Waltham, MA, United States). After adding barcode adaptors using the Ion Xpress Barcode Adaptors 1–16 Kit (Thermo Fisher Scientific), we purified the barcoded DNA using Agencourt AMPure XP (Beckman Coulter, Brea, CA, United States) and then combined 10 to 11 fragments. Next, we constructed a template DNA library on Ion Chef using the Ion 530 kit and 530 chip (Thermo Fisher Scientific), where we conducted sequencing by the Ion GeneStudio S5 sequencer (Thermo Fisher Scientific), according to the manufacturer’s protocols.

### Data analysis

To remove barcode and low-quality sequences, we processed the generated fastq files using the CLC Genomic Workbench ver. 21 (Qiagen, Aarhus, Denmark). Noting that, low-quality sequences were defined with <100 bases, maximum ambiguities of 2 and homopolymers >6 bases. Then, we assembled the trimmed reads *de novo* to contigs under the default conditions (minimum contig length, 1,000 bases; mismatch cost, 2; insertion cost, 3; detection cost, 3; length fraction, 0.5; similarity fraction, 0.8; global alignment, yes). Accordingly, the assembled files were subjected to the DFAST programme^1^ (Tanizawa et al., 2018; accessed on July 1, 2022) for genomic annotation. Simultaneously, the total assemblies were also subjected to each of the following: MLST 2.0 programme^2^ (Larsen et al., 2012; accessed on June 27, 2022) to assign *in silico* sequence types (STs); ResFinder programme ver. 4.1^3^ (Bortolaia et al., 2020; accessed on June 25, 2022) to detect acquired genes and/or chromosomal mutations mediating antimicrobial resistance; and CSI Phylogeny^4^ (Kaas et al., 2014; accessed on June 21, 2022) to generate the SNP-based phylogenomic tree under the following conditions: minimum depth at SNP positions, 10; relative depth at SNP positions, 10; minimum distance between SNPs (prune), 10; minimum SNP quality, 30; minimum read mapping quality, 25; and minimum Z-score, 1.96.

### Antibiotic susceptibility testing

The sequenced *C. jejuni* isolates (*n* = 111) were used to examine their susceptibility against three antibiotics, namely, ciprofloxacin (CPFX), tetracycline (TC) and ampicillin (ABPC) *via* disc diffusion test using Sensi-Disc (Becton Dickinson) according to the guidelines of the Clinical and Laboratory Standard Institute (CLSI, 2015).

### Statistical analysis

The statistical differences of the bacterial counts between different groups arranged by the slaughter age of chicken (<75 days and > 75 days) and the season (spring, summer, and fall; because of less sample numbers, the bacterial count data in winter was removed from the seasonal comparison) were calculated using the Mann–Whitney U test and the prevalence ratios of the AMR-related genes between the two groups (slaughter age at >75 days or < 75 days) were comparatively analysed using Pearson’s chi-squared test; the level of significance was set to *p* < 0.05.

## Results

### Quantitative detection of thermotolerant *Campylobacter* spp. in fresh chicken meats retailed in Japan

Overall, *Campylobacter* spp. were detected in 254 samples (49.8%), in which 27 (5.3%), 120 (23.5%), 64 (12.5%) and 43 (8.4%) samples were contaminated with *Campylobacter* spp., respectively, at the following ranges: 0.70–0.99 logCFU/g, 1.00–1.99 logCFU/g, 2.00–2.99 logCFU/g and >3.00 logCFU/g, respectively (Table 1). In total, the mean ± standard deviation (SD) and the maximum reach of *Campylobacter* counts were 1.15 ± 1.03 logCFU/g and 4.27 logCFU/g, respectively (Table 1).

**Table 1:** Quantitative detection of *Campylobacter* spp. from retail chicken meats at 5 distinct regions in Japan between June 2019 and May 2021.

Category	No. sample	Sample distribution by *Campylobacter* count (logCFU/g; %)								Prevalence rate of >3 logCFU/g
		ND^1^	0.70–0.99	1.00–1.99	2.00–2.99	3.00–3.99	4.00–4.99	Max	Means ± SD	
Region^2^										
A	20	18 (90.0%)	1 (5.0%)	1 (5.0%)	0 (0.0%)	0 (0.0%)	0 (0.0%)	1.00	0.40 ± 0.16	0.0%
B	295	129 (43.7%)	8 (2.7%)	72 (24.4%)	53 (18.0%)	31 (10.5%)	2 (0.7%)	4.27	1.36 ± 1.12	10.8%
C	74	37 (50.0%)	7 (9.5%)	20 (27.0%)	10 (13.5%)	0 (0.0%)	0 (0.0%)	2.78	0.94 ± 0.75	0.0%
D	24	16 (66.7%)	3 (12.5%)	4 (16.7%)	0 (0.0%)	1 (4.2%)	0 (0.0%)	3.62	0.77 ± 0.82	4.2%
E	97	56 (57.7%)	9 (9.3%)	22 (22.7%)	1 (1.0%)	9 (9.3%)	0 (0.0%)	3.88	0.91 ± 0.94	9.3%
Season^3^										
Spring	86	43 (50.0%)	4 (4.7%)	29 (33.7%)	5 (5.8%)	5 (5.8%)	0 (0.0%)	3.88	0.98 ± 0.88	5.8%
Summer	263	147 (55.9%)	10 (3.8%)	50 (19.0%)	37 (14.1%)	19 (7.2%)	0 (0.0%)	3.78	1.10 ± 1.02	6.8%
Fall	143	57 (39.9%)	11 (7.7%)	37 (25.9%)	20 (14.0%)	16 (11.2%)	2 (1.4%)	4.27	1.39 ± 1.12	12.6%
Winter	18	9 (50.0%)	2 (11.1%)	4 (22.2%)	2 (11.1%)	1 (5.6%)	0 (0.0%)	3.02	0.99 ± 0.87	5.6%
Slaughter age^4^										
<75 D	418	181 (43.3%)	23 (5.5%)	108 (25.8%)	63 (15.1%)	41 (1.0%)	2 (0.5%)	4.27	1.29 ± 1.08	10.3%
>75 D	92	75 (81.5%)	4 (4.3%)	12 (13.0%)	1 (1.1%)	0 (0.0%)	0 (0.0%)	2.18	0.52 ± 0.41	0.0%
Total	510	256 (50.2%)	27 (5.3%)	120 (23.5%)	64 (12.5%)	41 (8.0%)	2 (0.4%)	4.27	1.15 ± 1.03	8.4%

1 ND, not detected (<0.7 logCFU/g).

2 Regions A to E, shown in Supplementary Figure 1.

3 Spring, April to June; Summer, July to September; Fall, October to December; Winter, January to March.

4 The source chickens of meat products are categorized by slaughter age (<75 days or > 75 days of slaughter).

Seasonal comparison indicated the highest bacterial counts in the samples taken in fall (October to December, means of 4.27 logCFU/g) compared with those collected in spring (April–June, means of 3.88 logCFU/g) and summer (July–September, means of 3.78 logCFU/g), respectively (Table 1; Supplementary Figure 2A).

The comparison by slaughter age of chicken revealed that 56.7% (237/418) of meat samples from young broilers (slaughtered at <75 days old) were *Campylobacter*-positive with a mean ± SD of 1.29 ± 1.08 logCFU/g, which was significantly higher than the remaining 92 samples from older-aged chicken (slaughtered at >75 days old), which showed *Campylobacter* positivity at 18.5% (17/92) with a mean ± SD at 0.52 ± 0.41 logCFU/g (*p* < 0.01; Table 1; Supplementary Figure 2B).

Region comparison was excluded from the quantitative analysis because the sample numbers were not normalised between the five regions, as presented in Table 1.

Overall, we could show the current status of *Campylobacter* contamination levels in the chicken meat products retailed in Japan, in association with the slaughter age and season.

### Genomic characterisation of *Campylobacter jejuni* from the retailed chicken meat

Throughout the quantitative detection of *Campylobacter* spp., a total of 218 *C. jejuni* and 26 *C. coli* were finally isolated from 52.4% (244/466) of the samples in regions B, C and E (Table 1). We excluded *C. coli* for further characterization because its numbers were relatively scarce compared with *C. jejuni*. Among *C. jejuni*, 111 representative isolates (68 from chicken slaughtered at <75 days old and 43 at >75 days old) were arbitrarily selected. Thereby, a WGS was designed using the Ion GeneStudio S5 sequencing platform. The sequence read numbers ranged from 1,352,730 to 2,774,038 with an average read length ranging from 205 to 279 bases, in which *de novo* assembled genome statistics were at 23–92 contigs. The annotation of these assemblies using the DFAST programme identified 1,493–2,215 coding sequences (CDSs), 31–43 rRNAs, 1–2 tRNAs and coverage ratio of 87.1–93.5%, respectively (Supplementary Table 1).

#### MLST profiles

*In silico* MLST analysis classified the 111 *C. jejuni* isolates into 63 STs, where 45 STs were categorized in 12 clonal complexes (CC) and 9 of the remaining 18 STs were novel STs. Among the CCs, ST-21CC was the most predominant (24.3%, 27/111), followed by ST-354CC (13.5%, 15/111) and then ST-45CC (9.9%, 11/111). ST-22CC, ST-52CC and ST-607CC were detected only in the chicken meats slaughtered at <75 days old, whereas ST-353CC was detected only in the chicken meats slaughtered at >75 days old. Within ST-21CC that generally showed a broad range of host adaptation (Woodcock et al., 2017), ST-50 (*n* = 9), ST-21 (*n* = 2) and ST-9535 (*n* = 2) commonly originated from chicken meats slaughtered at <75 days old, whereas ST-11191 (*n* = 5), ST-4526 (*n* = 3) and ST-9776 (n = 2) were detected only in older slaughtered chicken meats (>75 days old). A total of 14 *C. jejuni* isolates from highly contaminated broiler chicken meat samples (>3.0 logCFU/g) were classified into ST-21CC (ST-50, *n* = 9), ST-22CC (ST-22, *n* = 2) and ST-45CC (ST-137, *n* = 1; ST-1326, *n* = 1; ST-3456, *n* = 1; Table 2).

**Table 2:** MLST and antimicrobial-resistance profiles of 111 representative *C. jejuni* isolates from chicken meat samples retailed in Japan.

Clonal complex (CC)	No. isolate	ST^2^	No. isolate	Slaughter age of chicken^3^	Prevalence of AMR-related gene			No. isolate exhibiting resistance to		
					*gyrA* (T86I)	*tetO*	*bla*_OXA-61_	CPFX	TC	ABPC
ST-21 CC	27	21	2	Y (2)	2/2	2/2	2/2	2	2	2
		50	9	Y (9)	0/9	0/9	0/9	0	0	0
		4,526	3	O (3)	3/3	3/3	3/3	3	3	3
		6,709	1	Y (1)	0/1	0/1	1/1	0	0	1
		8,132	1	O (1)	0/1	1/1	1/1	0	1	1
		9,535	2	Y (2)	1/2	1/2	2/2	1	1	2
		9,776	2	O (2)	0/2	0/2	2/2	0	0	2
		**11,191**	5	O (5)	0/5	0/5	5/5	0	0	5
		**11,572**	1	Y (1)	0/1	0/1	1/1	0	0	1
		**11,574**	1	Y (1)	0/1	0/1	0/1	0	0	0
ST-354 CC	15	354	8	O (5), Y (3)	4/8 (O(4))	0/8	0/8	4	0	0
		653	1	Y (1)	1/1	1/1	0/1	1	1	0
		1723	1	O (1)	0/1	0/1	0/1	0	0	0
		6,196	2	O (1), Y (1)	0/2	0/2	0/2	0	0	0
		10,010	1	Y (1)	0/1	0/1	0/1	0	0	0
		**11,347**	1	Y (1)	0/1	0/1	0/1	0	0	0
		**11,352**	1	Y (1)	0/1	0/1	0/1	0	0	0
ST-45 CC	11	11	1	Y (1)	0/1	0/1	0/1	0	0	0
		45	3	Y (3)	2/3	3/3	2/3	2	3	2
		137	1	Y (1)	0/1	0/1	1/1	0	0	1
		538	1	Y (1)	0/1	1/1	1/1	0	1	1
		1,326	1	Y (1)	0/1	0/1	0/1	0	0	0
		3,456	1	Y (1)	1/1	1/1	0/1	1	1	0
		9,295	1	Y (1)	1/1	1/1	0/1	1	1	0
		**11,192**	1	O (1)	0/1	1/1	0/1	0	1	0
		**11,302**	1	O (1)	0/1	0/1	0/1	0	0	0
ST-464 CC	10	4,108	1	O (1)	0/1	0/1	0/1	0	0	0
		4,389	3	O (2), Y (1)	1/3 (O)	0/3	0/3	1	0	0
		6,477	1	O (1)	0/1	0/1	0/1	0	0	0
		6,704	3	Y (3)	0/3	0/3	1/3	0	0	1
		**11,024**	1	O (1)	1/1	0/1	0/1	1	0	0
		**11,186**	1	Y (1)	0/1	0/1	1/1	0	0	1
ST-443 CC	6	440	4	O (2), Y (2)	1/4 (Y)	0/4	4/4	1	0	4
		6,512	2	Y (2)	0/2	0/2	2/2	0	0	2
ST-22 CC	3	22	2	Y (2)	2/2	0/2	2/2	2	0	2
		567	1	Y (1)	0/1	0/1	1/1	0	0	1
ST-353 CC	3	8,133	2	O (2)	0/2	0/2	2/2	0	0	2
		10,013	1	O (1)	0/1	0/1	0/1	0	0	0
ST-48 CC	2	918	2	O (1), Y (1)	0/2	0/2	2/2	0	0	2
ST-52 CC	2	52	2	Y (2)	2/2	0/2	0/2	2	0	0
ST-460 CC	1	**11,190**	1	O (1)	0/1	0/1	0/1	0	0	0
ST-206 CC	1	2,282	1	O (1)	0/1	0/1	1/1	0	0	1
ST-607 CC	2	3,646	1	Y (1)	0/1	0/1	0/1	0	0	0
		**11,569**	1	Y (1)	0/1	0/1	0/1	0	0	0
UA^1^	28	407	1	Y (1)	1/1	1/1	1/1	1	1	1		
		468	1	Y (1)	0/1	0/1	0/1	0	0	0		
		922	1	Y (1)	0/1	1/1	1/1	0	1	1		
		1972	2	O (2)	0/2	0/2	0/2	0	0	0		
		4,324	1	Y (1)	0/1	0/1	1/1	0	0	1		
		4,325	1	Y (1)	0/1	0/1	1/1	0	0	1		
		6,085	1	O (1)	0/1	1/1	0/1	0	1	0		
		8,071	4	O (1), Y (3)	4/4	1/4 (Y)	4/4	4	1	4		
		8,287	1	Y (1)	0/1	0/1	0/1	0	0	0		
		**11,187**	1	O (1)	0/1	1/1	0/1	0	1	0		
		**11,189**	1	O (1)	0/1	0/1	0/1	0	0	0		
		**11,194**	1	O (1)	0/1	1/1	0/1	0	1	0		
		**11,195**	2	Y (2)	0/2	2/2	0/2	0	2	0		
		**11,342**	1	Y (1)	0/1	0/1	1/1	0	0	1		
		**11,343**	1	O (1)	1/1	1/1	1/1	1	1	1		
		**11,344**	6	Y (6)	0/6	6/6	0/6	0	6	0		
		**11,349**	1	Y (1)	0/1	1/1	0/1	0	1	0		
		**11,570**	1	Y (1)	0/1	0/1	0/1	0	0	0
Total	111	–	111	Y (68)	17/68 (25.0%)	22/68 (32.4%)	28/68 (41.2%)	17	22	28
				O (43)	11/43 (25.6%)	9/43 (20.9%)	19/43 (44.2%)	11	9	19
				Y+ O (111)	28/111 (25.2%)	31/111 (27.9%)	47/111 (42.3%)	28	31	47
*p*-value (Y vs. O)^4^					0.918	0.146	0.757	–	–	–
*P*-value (CCs)^5^					0.322	<0.01	<0.01	–	–	–

1 UA, Unassigned to any clonal complexes.

2 Sequence type (ST). Novel STs are in bold and STs originated from highly contaminated samples (>3.0 logCFU/g) are underlined.

3 Y, young broiler chicken slaughtered at <75 days age; O, chicken slaughtered at > 75 days age.

Numbers of isolates are shown in the parenthesis.

4 Difference for the prevalence of AMR-related genes between the groups Y and O calculated by Pearson’s chi-squared test.

5 Difference for the prevalence of AMR-related genes between CCs calculated by Pearson’s chi-squared test.

#### Antimicrobial resistance profiles

ResFinder predicted that 42.3% (47/111) of *C. jejuni* isolates harboured *bla_OXA-61_* gene, 27.9% (31/111), *tetO* gene; and 25.2% (28/111), *gyrA* mutated at T86I (Table 2). The antimicrobial susceptibility testing resulted in the complete agreement between the presence of three antimicrobial resistance (AMR)-related genes and resistance phenotype against the three antimicrobials among the sequenced isolates (Table 2). 24 isolates (18 isolates were from meats slaughtered at <75 days old and 6 from those at >75 days old) showed resistance to more than two antimicrobials, in which 14 isolates were resistant to both TC and CPFX (Table 2). The prevalence of three AMR-related genes did not exhibit statistical association with the slaughtered age of chicken (Table 2).

#### Association between the MLST and AMR profiles

Combined with the MLST profiles, *gyrA* (T86I) was detected in 22/83 (26.5%) isolates belonging to 7 CCs [(ST-21CC (6/27), ST-22CC (2/3), ST-45CC (4/11), ST-52CC (2/2), ST-354CC (5/15), ST-443CC (1/6) and ST-464CC (2/10)] and 6/28 (21.4%) isolates unassigned (UA) to any CCs with no significant difference between CCs (*p* = 0.32; Table 2).

Alternately, *tetO* was detected in 15/83 (18.1%) isolates belonging to 3 CCs [ST-21 CC (7/27), ST-45 CC (7/11) and ST-354CC (1/15)], and 16/28 (57.1%) of UA isolates with significant difference between CCs (*p* < 0.01; Table 2).

Similarly, *bla_OXA-61_* was detected in 37/83 (44.6%) isolates belonging to 8 CCs [ST-21CC (17/27), ST-22CC (3/3), ST-45CC (4/11), ST-48 CC (2/2), ST-206CC (1/1), ST-353CC (2/3), ST-443CC (6/6) and ST-464CC (2/10)], and 10/28 (35.7%) of UA isolates with significant differences between CCs (*p* < 0.01; Table 2).

The multidrug-resistant isolates were shown in 24/111 (21.6%) isolates, 13 of which were belonged to ST-21CC (8/27) and ST-45CC (5/11), although there were no significant differences between CCs (*p* = 0.33; Table 2).

In summary, we detected the MLST and AMR profiles of 111 representative *C. jejuni* from chicken meats retailed in Japan, with partial links to slaughter age of chicken and contamination levels.

### Phylogenetic diversity of *Campylobacter jejuni* isolates from chicken meat samples

**Figure 1:**
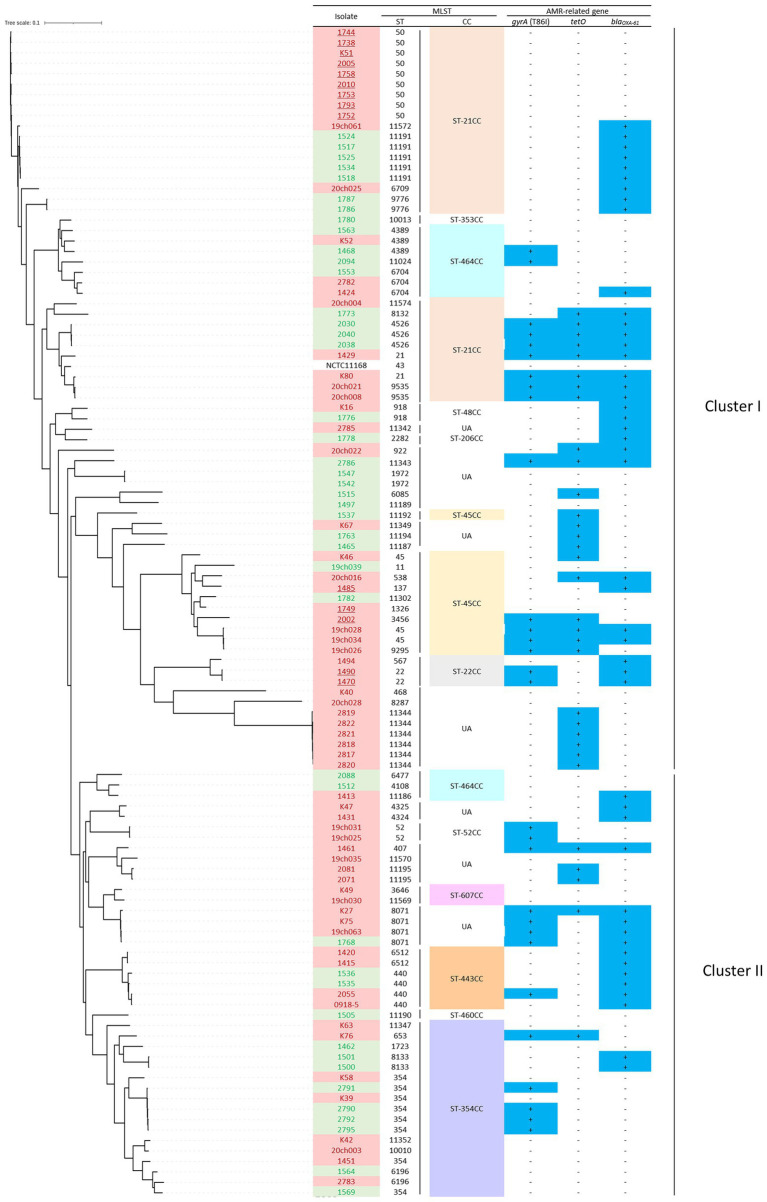
The SNPs-based phylogenetic tree of 111 *C. jejuni* isolates from fresh chicken meats retailed at regions B, C and E in Japan, with MLST (ST and CC) and AMR profiles (possession (+/−) of three AMR-related genes). Each isolate is highlighted in red (slaughter age of <75 days) or green (slaughter age of >75 days). *C. jejuni* isolates of highly contaminated samples (>3.0 logCFU/g) are underlined. CCs in which >3 isolates included are highlighted with different colours. The prevalence of AMR-related gene is shown as “+” in dark blue-highlight.

To examine the possible association of the bacterial genomic traits with the slaughtered age of chicken and bacterial contamination levels, a single nucleotide polymorphism (SNP)-based phylogenetic tree was constructed, which was broadly divided into two clusters (I and II); the first consisted of 42 young broiler-originating isolates (red) and 28 older isolates (green), whereas cluster II consisted of 26 young (red) and 15 older ones (green; Figure 1), with no apparent statistical phylogenic bias based on the chicken slaughter age.

Within the MLST profiles, seven CCs (i.e., ST-21CC, -22CC and -45CC) belonged to cluster I and six (i.e., ST-354CC, ST-443CC and ST-464CC) to cluster II (Figure 1). Notably, 14 isolates that originated from samples exhibiting >3.0 logCFU/g of contamination were all distributed in cluster I and none in cluster II (isolates underlined in Figure 1).

Thus, these data indicated that the *C. jejuni* genotypes in cluster I were partly associated with highly contaminated chicken meat samples.

## Discussion

*Campylobacter* in poultry meat is ranked as the leading pathogen–food combination to cause health risks; therefore, it negatively impacts the national economy (Kaakoush et al., 2015; Batz et al., 2021). The ratio of highly contaminated samples (>3.0 logCFU/g) in our data was estimated at 8.4%, which was seemingly almost similar or less, compared with some recent reports from other countries; for example, Asuming-Bediako et al. reported that 12.7% (7/55) of the chicken meats retailed in Ghana were >3.0 logCFU/g (Asuming-Bediako et al., 2022). Moreover, 18.7% (59/315) of chicken meats retailed in Australia were contaminated with >2.0 logCFU/g (Habib et al., 2019).

In Japan, most of the chicken meats are retailed as portion fillets, not as whole carcass, which suggested that there might be differences due to processing. Therefore, a nationwide baseline surveillance at both slaughter and retail stages could better estimate the stage-to-stage kinetics of the bacterial contamination levels, thereby providing the QMRA data. Nevertheless, to the best of our knowledge, this is the first study to demonstrate the quantitative distribution of thermophilic *Campylobacter* spp. in chicken meat products retailed across Japan.

To date, it is likely that human campylobacteriosis occurred more frequently in hot seasons in summer and/or fall compared with cold seasons, as proven in Denmark (Nielsen et al., 2013), France (Bessède et al., 2014), United States (Williams et al., 2015; Sher et al., 2021), Switzerland (Baumgartner and Felleisen, 2011). Likewise, in Japan, the greater proportions of campylobacteriosis were reported in summer and fall based on the foodborne disease statistics report [Ministry of Health, Labor and Welfare of Japan (MHLW), 2021]. Our data indicated greater bacterial counts in chicken meats retailed in fall than spring and summer, which seemed to be in agreement with the foodborne statistics shown above. Several factors considerably affect the seasonal variation of bacterial counts, one of which seemed to be linked to the geography-associated atmospheric variations. For example, the mean temperature which is identified as one of the major environmental drivers for the campylobacteriosis occurrence (Cousins et al., 2020), was 22.5°C in region A and 28.4°C in region B, on August 2019, according to the Japanese meteorological agency.^5^ Continued surveillance at different regions with atmospheric information might improve our understanding behind the effects of temperature and/or geographical differences in the quantitative distribution of *Campylobacter* spp. in meat chickens on the farm. Although this study did not include human isolates, future continued monitoring and characterization of *Campylobacter* spp. from chicken meats together with human isolates would further unveil the seasonal dynamics of *Campylobacter*.

In this relation, we previously demonstrated that long-term grow-out in laying hens affects the *C. jejuni* colonization fitness, which is associated with altered gut microbiota and lipid compositions (Asakura et al., 2021). Considering the fact that *C. jejuni* cooperated and competed with diverse commensal microbiota, thereby becoming part of the gut microbial community (Han et al., 2017; Sakaridis et al., 2018), our data associated with the slaughter age also indicated that long-term feeding of chicken might reduce the colonising numbers of *Campylobacter* in the chicken gut.

The increasing availability of WGS datasets has significantly enhanced the analyses of bacterial population structure and diversity (Sheppard and Maiden, 2015). Several studies have attempted to trace the source of infection by identifying the genomic elements (SNPs and genes) segregated by the host (Weis et al., 2016; Thépault et al., 2018; Berthenet et al., 2019). Moreover, the WGS analysis allows the detection and characterisation of antimicrobial resistance of *Campylobacter* isolates (Whitehouse et al., 2018; Meistere et al., 2019).

Among the STs associated with high level of contamination in poultry meats, it is noteworthy that multiple ST-50 isolates, in particular, were identified, although chicken were slaughtered and processed in different facilities at different time points. This genotype is known as one of the major host generalists (Woodcock et al., 2017) and linked to human infection (Ramonaite et al., 2017). However, we could not conclude the association between the specific clonal complexes and contamination levels in the chicken meats, because the parts of ST-45CC and ST-22CC showed association with high level contamination. In addition, it was uncertain why *C. jejuni* isolates belonged to ST-21CC were phylogenetically split into two subclusters within cluster I. Considering the strain-to-strain variations of virulence property even in ST-21CC, which were assessed by multiple infection models (Humphrey et al., 2015), these strain’s virulence should be further assessed using these infection models in our future study.

Contrarily, several STs classified into cluster II originated from samples with less bacterial counts. An *in vitro* cell invasion property-based dose–response modelling was recently proposed (Abe et al., 2021), and it would be useful to use this approach to investigate possible differences in the virulence-associated characteristics clusters I and II. In addition, ST-4526, which previously thrived in Japan in human and chicken (Asakura et al., 2012), was isolated from meats of chicken slaughtered >75 days age. Considering that, this genotype could exhibit an increased colonisation fitness in the chicken’s gut (Asakura et al., 2013), the reason for which it was isolated from chicken meats being fed for long periods.

A recent study demonstrated that compared with infected patients with *C. jejuni* exhibiting TC resistance, those with CPFX resistance were more likely to be hospitalised during fall or summer seasons in the United States (Rodrigues et al., 2021). Our data demonstrating the widespread of genes conferring resistance to TC (*tetO*), β-lactam (*bla_OXA-61_*) and fluoroquinolone (*gyrA* T86I), were shown particularly among the isolates belonging to ST-21CC and ST-45CC, which are among the host generalist genotypes, thus transmissive between chicken and humans (Sheppard et al., 2014; Mourkas et al., 2020). These data suggest the necessity to monitor these CCs for antimicrobial resistance in both chicken and humans. Indeed, there were significant differences of the prevalence of *tetO* and *bla_OXA-61_* genes between CCs. Moreover, a recent study demonstrated the increased conjugative efficiency of *tetO*-carrying plasmids between *Campylobacter* strains at 42°C (resembling poultry reservoir) compared with 37°C (Cuevas-Ferrando et al., 2020), which further highlighted the needs to regulate the invasion of these CCs into poultry farm in order to decrease the chance of spreading TC-resistance. The stable kinetics of fluoroquinolone and TC resistance were observed in *C. jejuni* from farmed animals and humans in the United States and United Kingdom (van Vliet et al., 2022) and the association of antimicrobial resistance of *C. jejuni* in broilers with their use at farm was recently investigated (Tenhagen et al., 2021), which showed a positive association between use of tetracycline and erythromycin and subsequent resistance in *Campylobacter* but no such link was found for aminoglycosides. In relation to the latter, for example 77.1% of the total amounts of oral enrofloxacin distributed in Japan (1,829.4 kg) was used in broilers until 7 days before slaughter in 2019 [National Veterinary Assay Laboratory (NVAL), 2019]. Consequently, a spatiotemporal comparative monitoring of the antimicrobial resistance of this pathogen to commonly used antibiotics in farmed animals, foods and humans is essential, where the approach of reducing antimicrobial use in farms would be advised from a healthcare viewpoint.

## Conclusion

In this research study, we validated the current status of the *Campylobacter* contamination levels in retail chicken meats in Japan. Our data proposed the necessity to further reduce the contamination levels similar to European countries, which are attempting to reduce the highly contaminated chicken meats at retail (>3.0 logCFU/g) to zero (European Commission, 2017). Combining the different parameters affecting the occurrence and contamination levels of this pathogen (age of the slaughtered chicken meats and seasonal and bacterial phylogenetic variations) as well as the MLST-based source attribution study (Cody et al., 2019) might help QMRA to develop adequate control measures to reduce bacterial contamination in chicken meats, thereby leading to the reduction of the incidence of human campylobacteriosis through a continuous baseline surveillance with quantitative testing and bacterial genome characterization through the food chain.

## Data availability statement

The datasets presented in this study can be found in online repositories. The names of the repository/repositories and accession number(s) can be found in the article/Supplementary material.

## Author contributions

HA conceptualized, designed, supervised the experiments, and wrote the original draft. SY, KY, JK, HN, KA, YS, TI, and RN conducted experiments and data analysis. HA, KY, JK, and HN conducted a writing review. All authors contributed to the article and approved the submitted version.

## Funding

This research was supported in part by a grant from Ministry of Health, Labour and Welfare of Japan (grant number 20KA1007) and a grant from the Food Safety Commission of Japan (grant number JPCAFSC20222202).

## Conflict of interest

The authors declare that the research was conducted in the absence of any commercial or financial relationships that could be construed as a potential conflict of interest.

## Publisher’s note

All claims expressed in this article are solely those of the authors and do not necessarily represent those of their affiliated organizations, or those of the publisher, the editors and the reviewers. Any product that may be evaluated in this article, or claim that may be made by its manufacturer, is not guaranteed or endorsed by the publisher.

## Footnotes

^1^ https://dfast.ddbj.nig.ac.jp/

^2^ https://cge.cbs.dtu.dk/services/MLST/

^3^ https://cge.cbs.dtu.dk/services/ResFinder/

^4^ https://cge.cbs.dtu.dk/services/CSIPhylogeny/

^5^ www.data.jma.go.jp
