# Supplementary material for: Quantitative detection and genetic characterization of thermotolerant Campylobacter spp. in fresh chicken meats at retail in Japan
Source: Front Microbiol. 2022 Oct 10;13:1014212. doi: 10.3389/fmicb.2022.1014212 (PMC9589359; doi:10.3389/fmicb.2022.1014212)
Supplement: Supplementary file 1 [file Data_Sheet_1.DOCX]

Supplementary Materials


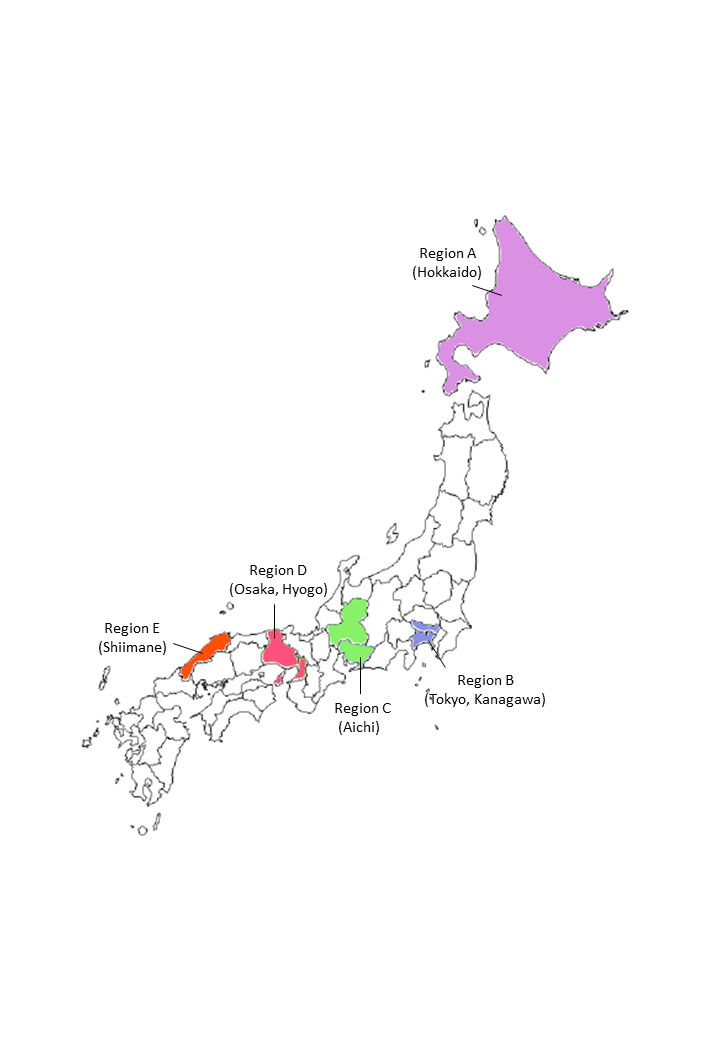


**Supplementary Figure 1.** Geographical information of the sampling regions in Japan.


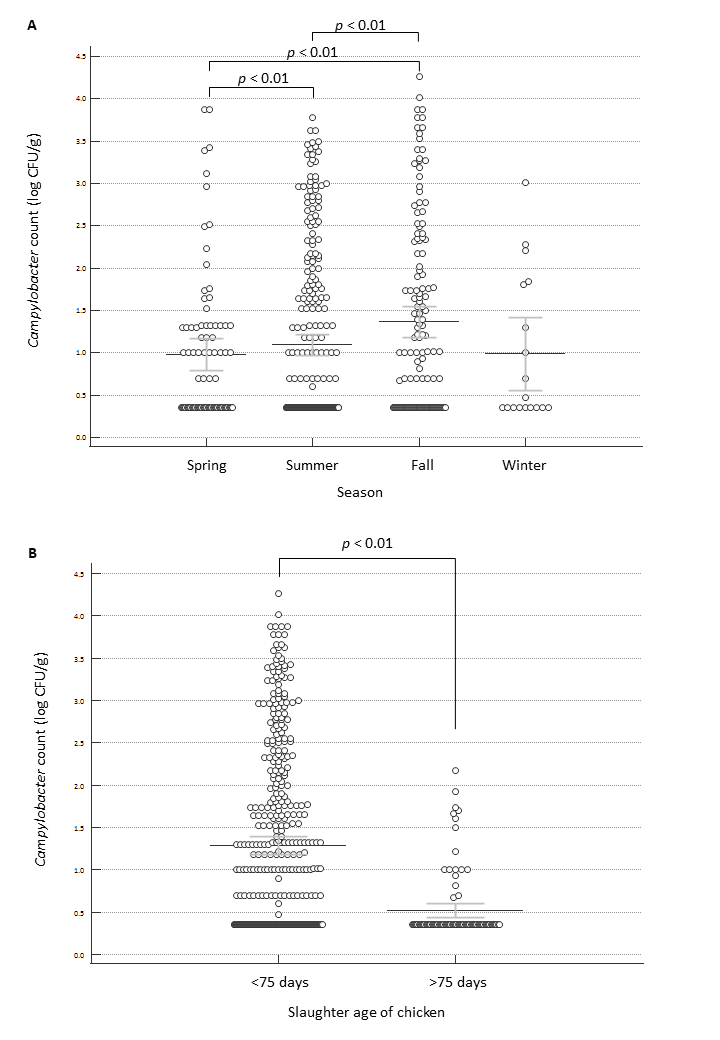


**Supplementary Figure 2.** Comparison of *Campylobacter* counts on chicken meat samples by season (A) and slaughter age of chicken (B).

**Supplementary Table 1.** Statistics of whole genome sequencing analysis of *C. jejuni* isolates from chicken meat samples.

^*1^ *C. jejuni* isolates originated from highly contaminated samples (>3.0 logCFU/g) are underlined.

^*2^ *de novo* assembled information is acquired by DFAST program.
